# Change of direction and Repeated Sprint Ability with and without ball performance in young soccer players: a comparison across different age-categories

**DOI:** 10.7717/peerj.20691

**Published:** 2026-02-02

**Authors:** Mehdi Ben Brahim, Farjana Akter Boby, Ariadna Hernaiz-Sánchez, Hussain Yasin, Alejandro Sal-de-Rellán

**Affiliations:** 1 Health and Physical Education Department, Prince Sultan University, Riyadh, Saudi Arabia; 2 Department of Physical Education & Sports Science, Daffodil International University, Dhaka, Bangladesh; 3 Department of Education and Educational Innovation, Faculty of Law, Education and Humanities, Universidad Europea de Madrid, Madrid, Spain

**Keywords:** Agility testing, Physical performance, Physical development, Team sport, Football

## Abstract

**Background:**

This study aimed to investigate age-related differences in anthropometric characteristics, change of direction (COD) and repeated sprint ability (RSA) performance, with and without ball control, in elite soccer players from U17, U19, and U23 categories.

**Methodology:**

Seventy-two male players (age: 18.9 ± 2.23 years; height: 1.72 ± 0.08 m; body mass: 71.7 ± 5.04 kg; body mass index (BMI): 24.3 ± 2.61 kg/m^2^) from three professional soccer clubs were assessed (U17 = 24; U19 = 24; U23 = 24). After a two-month period of regular training and competition, anthropometric measures (height, body mass, body mass index) were recorded. In addition, players completed the New Multi-Change of Direction Agility Test (NMAT) and the Bangsbo RSA test, both performed with and without a ball. Testing was standardized for familiarization, warm-up, and environmental conditions.

**Results:**

U23 players were taller and heavier than U17 and U19 players, and they showed superior COD performance without the ball compared to U17, whereas no statistically significant differences were found in COD with ball or RSA performance across age groups. Correlation analyses revealed moderate associations between anthropometric variables and COD performance (*r* =  − 0.35 to −0.24), while higher BMI values were related to slower agility times (*r* = 0.24–0.26).

**Conclusions:**

Age-related anthropometric characteristics were associated with better COD performance without the ball, whereas COD with ball and RSA performance appear less age-dependent and more influenced by training specificity. These findings highlight the importance of incorporating technical COD drills and RSA training early in player development to align physical and technical progression.

## Introduction

Soccer is a high-intensity intermittent team sport that requires players to perform a wide variety of physical actions, including accelerations, decelerations and changes of direction (COD) alternated with periods of lower effort (Nobari et al., 2023; Pons et al., 2021). A professional soccer player often covers 10 to 12 km per match (Modric et al., 2023). Moreover, players often cover roughly 418–568 m every match while sprinting at a high velocity (*i.e.,* 21–24 km h^−1^) (Rey et al., 2023) and execute around 100–150 accelerations (Varley & Aughey, 2012). Additionally, soccer players must consistently execute maximum or near-maximal short-duration sprints (1–7 s) with minimal recovery periods (Girard, Mendez-Villanueva & Bishop, 2011; Kyles et al., 2023) completing repeated sprint ability (RSA) and COD performance key components of soccer-specific fitness (McBurnie et al., 2022; Rouissi et al., 2017).

While several studies suggest that the peak performance age in soccer is approximately 26 years (Kalén et al., 2019; Sal-de-Rellán et al., 2023), the increasing integration of young athletes (16–23 years) into professional squads has shifted attention towards understanding performance demands in youth stages. These younger players tend to cover a greater number of sprints (*i.e.,* 19.3 ± 7.8) and number of fast runs (*i.e.,* 59.2 ± 15.6) than older soccer players (Sal de Rellán-Guerra et al., 2019) and cover longer distances during matches at high speed (*i.e.,* 259–282 ± 102 m), very high speed (*i.e.,* 541–568 ± 238 m) and sprinting (*i.e.,* 281–285 ± 161 m) (García-Calvo et al., 2023; Rey et al., 2023). Additionally, the coefficient of variation for high-intensity actions is lower in players under 25 than in those over 33, indicating a more consistent performance (Lorenzo-Martínez, Rey & Padrón-Cabo, 2020). Specifically, elite youth soccer players perform 305 ± 50 CODs on average (Morgan et al., 2022).

COD ability, both with and without the ball, is a critical performance factor in soccer, enabling players to effectively react to rapidly changing game scenarios such as evading opponents, intercepting passes, or adjusting defensive positioning (Borges et al., 2023; Plakias et al., 2024; Praça et al., 2020). The inclusion of ball control during COD actions imposes additional technical, physical, and cognitive demands (Morral-Yepes et al., 2023) and comparing performance in both conditions provides insights into the interaction between physical capacity and technical skill (Lerche et al., 2024; Trecroci et al., 2020). The developmental trajectory of COD performance is influenced by factors such as anthropometric, neuromuscular coordination, technical skill acquisition, and exposure to structured training (Arede et al., 2019; Thieschäfer & Büsch, 2022). Existing literature also emphasizes the multifaceted nature of COD ability, which depends on strength, agility, power, motor coordination, and technical skills such as dribbling and ball control (Nimphius et al., 2016; Young, Dawson & Henry, 2015). For example, Young, McDowell & Scarlett (2001) demonstrated that dribbling adds physical and cognitive demands that affect overall agility. The New Multi-Change of Direction Agility Test (NMAT) replicates soccer-specific movement patterns and assesses COD performance with and without the ball (Ben Brahim, Bougatfa & Amri, 2013), providing insights into the integration of physical and technical demands in agility performance.

Similary, RSA is another key component of soccer performance, as it reflects a player’s capacity to perform short-duration maximal sprints with minimal recovery closely simulating the physical demands of competitive matches (Kyles et al., 2023). RSA tests have traditionally involved straight-line sprints of 15–40 m, but more recent protocols include changes of direction and technical actions to better replicate match scenarios (Impellizzeri et al., 2008; Martin et al., 2018). The Bangsbo RSA test is widely used in elite soccer due to its strong ecological validity, incorporating repeated efforts with short active recoveries to assess both anaerobic power and fatigue resistance (Bangsbo, 1994; Rampinini et al., 2007). Performance in RSA tests has been shown to correlate with critical physical qualities such as acceleration, agility, explosive leg power, and aerobic endurance (Rodríguez-Fernández et al., 2019; Spencer et al., 2011). It also varies depending on anthropometric factors. However, few studies have examined how RSA performance evolves across youth age categories (*e.g.*, U17, U19, U23), particularly under both technical (with ball) and non-technical (without ball) conditions. This limits the applicability of current evidence for the design of age appropriate, soccer specific training interventions.

Despite increasing research, there remains a lack of studies analyzing COD and RSA performance simultaneously under both ball and no-ball conditions across different age categories. Additionally, the influence of anthropometric characteristics on these abilities in youth soccer players is poorly understood. Therefore, this study aimed to investigate age-related differences in COD and RSA performance, with and without ball control, in soccer players from U17, U19, and U23 categories. Variations in anthropometric traits across these age groups were also examined. The NMAT and Bangsbo RSA tests were employed to provide a detailed assessment of physical and technical demands throughout a player’s development stages. Consequently, based on previous studies (Borges et al., 2023; Loturco et al., 2020; Silva et al., 2025), it is hypothesized that players in the U23 category would display superior performance in COD and RSA tests compared to the U17 and U19 categories, with the greatest differences expected in the no-ball condition, where technical constraints are minimal and physical/anthropometric/training factors may dominate.

## Materials & Methods

### Participants

The study was carried out in collaboration with three professional soccer clubs. Seventy-two Tunisian male soccer players who regularly competed nationally and internationally and were registered with the clubs’ first teams or their official youth academies (age: 18.9 ± 2.23 years; height: 1.72 ± 0.08 m; body mass: 71.70 ± 5.04 kg; body mass index (BMI): 24.3 ± 2.61 kg/m^2^) voluntarily participated in this study, divided into three distinct age categories: under-17 (U17) (age: 16.8 ± 0.44 years; height: 1.69 ± 0.08 m; body mass: 68.53 ± 5.05 kg; BMI: 24.1 ± 2.49 kg/m^2^), Under-19 (U19) (age: 18.3 ± 0.48 years; height: 1.70 ± 0.07 m; body mass: 73 ± 4.15 kg; BMI: 25.5 ± 2.70 kg/m^2^), and Under-23 (U23) (age: 21.8 ± 1.11 years; height: 1.78 ± 0.07 m; body mass: 73.56 ± 4.45 kg; BMI: 23.4 ± 2.28 kg/m^2^). Players were actively engaged in structured soccer training programmes and were recruited through purposive sampling. Inclusion criteria required players to be injury-free for at least six months prior to testing, actively involved in competitive soccer, limited sports for over seven days, and having taken part in 90% of the training sessions. Exclusion criteria included recent injuries or medical conditions that could affect performance. Players and parents/guardians were informed of the study’s procedures, potential risks/benefits, and both signed a written informed consent before starting the research. Participant confidentiality was maintained by anonymizing all data. In addition, the study was performed in accordance with the Declaration of Helsinki (2013). The study protocol was reviewed and approved by the Institutional Review Board at Prince Sultan University, Saudi Arabia (PSU IRB-2025-08-0239). Any potential risks associated with testing were minimized by ensuring the presence of trained personnel and medical staff during data collection.

### Measures

This cross-sectional study was conducted to evaluate the COD and RSA performance with and without a ball, among soccer players of different age groups. Data collection occurred over a two-month period during regular training and competition seasons to ensure ecological validity. During the week preceding the experiment, players familiarized themselves with the testing protocols. No specific training aimed at improving COD or RSA performance was conducted during this period. Morning training sessions lasted 60 min and were performed at the same time (*i.e.,* 9:30 a.m.), while the afternoon sessions lasted 90 min and were also performed at the same time (*i.e.,* 17:30 p.m.). All testing sessions were performed in the morning between 9:00 a.m. and 11:00 a.m. under similar environmental conditions to minimize circadian variability in performance. Testing took place outdoors on natural grass, and under similar environmental conditions (23–25 ° C), with the same sports clothes, and were issued by the same testers. Before testing, a general and specific warm-up routine was performed, involving 3-min of jogging, followed by 5-min of dynamic and ballistic stretching, and 7-min of progressive sprints and accelerations (Ben Brahim et al., 2023a; Ben Brahim et al., 2023b).

During the experimental period, all players followed a weekly standard training programme. Mondays were dedicated to regeneration sessions for all participants. On Tuesdays, players completed single daily sessions combining conditioning, technical, and tactical training with game simulations. Additional technical and tactical training sessions were conducted during the afternoon. Wednesday sessions focused on combined finishing drills with technical-tactical training and game simulations. Thursday’s training followed a similar structure to Tuesday’s, with conditioning and tactical training in the morning and game-based tactical training in the afternoon. Fridays emphasized conditioning and technical-tactical drills for all players, with recovery days every three weeks. Saturdays were reserved for technical and tactical training with game simulations, while Sundays were match days for all participants. Measurements were taken at the end of the eight weeks of standardized training (*i.e.,* week 9). Before assessment sessions, a general and specific warm-up routine was executed (Ben Brahim et al., 2023a; Ben Brahim et al., 2023b). On the first day of evaluation (Tuesday), new multi-change of direction agility tests with and without ball were conducted. The second day (Thursday) involved the Bangsbo RSA test with and without ball. Anthropometric measurements were taken in both sessions, and the mean of both measurements was used. To reduce the influence of uncontrolled variables, players were instructed to maintain their habitual lifestyle and normal dietary intake before and during the study.

### Design and procedures

#### Anthropometric measurements and age

Anthropometric data included height (m), body mass (kg), body mass index (BMI) (kg/m^2^), and age. Anthropometric values were measured for all participants on both measurement days. For each player, the anthropometric variables of height (in meters) and body mass (in kilograms) were measured. Height was measured to the nearest 0.1 cm using a stadiometer (Holtain Ltd., Crymych, United Kingdom). Body mass was collected to the nearest 0.1 kg using an electronic scale (Seca Instruments Ltd., Hamburg, Germany). BMI was calculated using the formula: Body mass*(Height/100)^−2^. Age was collected on the first day of the measurements.

#### Battery fitness test

*New multi-change of direction agility test (NMAT)*, as described in the literature by Ben Brahim, Bougatfa & Amri (2013). In this test, the players’ velocity during a 25 m agility course was measured using a photocell timing system (Cell Kit Speed Brower, USA). Each player began with a 2.5 m lateral displacement, returned the same distance to the starting point, and then performed a 2.5 m backward run followed by a 3 m forward sprint. The athlete then executed a 1 m directional change, continued with a 1.35 m linear sprint, and jumped over a 0.5 m barrier before finishing with a 5 m straight run to the final timing gate. Two trials were completed under each condition (without the ball: NMAT; with the ball: NMAT-B), separated by 5 min of passive recovery, and the best time was recorded.

*Bangsbo RSA test*, as described in the literature by Bangsbo (1994), evaluated repeated sprint ability over a 7 × 34.2-m course using the same set of photocell gates (Racetime2^®^, Microgate, Bolzano, Italy) which were positioned 0.4 m above the ground at both the starting point and at 34.2 m. Players started 50 centimeters behind the timing gate and sprinted 10-m before executing a right or left directional swing. The sprint continued to the finish line where another timing gate recorded completion time. Players then engaged in a 25-second active recovery, jogging back to the starting position. Verbal cues were provided at 10, 20, and 23 s, with a starting signal given at 25 s. This sequence was repeated for a total of seven sprints. The 7 × 34.2 m repeated sprint was executed at maximum speed, with verbal feedback given to each player throughout the recovery phase to enhance their preparedness for the subsequent sprint. The test concluded with the completion of the 7 × 34.2 m repeated sprints by each participant. The variables collected were RSA Best for both conditions: without the ball (Bangsbo) and with the ball (Bangsbo-B).

The order of the ‘with ball’ and ‘without ball’ conditions for both COD and RSA tests was randomized using a simple procedure to minimize potential order effects. Each participant drew a numbered card to determine the test sequence.

### Statistical analysis

The descriptive analysis was presented as mean ± standard deviation (SD). For the assumption of normality, the Shapiro–Wilk test was used, which confirmed that the data had a normal distribution, while the Levene test showed that the variance was homogeneous. A repeated measures analysis of variance (ANOVA) was applied to detect differences in the battery fitness test regarding each age group (*i.e.,* U17, U19, U23). Partial eta-squared (*η*^2^_*p*_) effect sizes (ES) for group interaction were calculated. Effect sizes were interpreted as follows: trivial (*η*^2^_*p*_ < 0.01), small (*η*^2^_*p*_ ≥ 0.01), medium (≥0.059), and large (≥0.138) (Cohen, 2013). When significant differences were observed, a *post hoc*’s with Bonferroni corrections was applied. Cohen’s d was used to calculate the effect size, using the following classification: trivial (<0.2), small (0.2–0.59), moderate (0.6–1.19), large (1.2–1.99), very large (2.0–3.99), and extremely large (≥4.0) (Hopkins et al., 2009). In addition, Pearson’s correlation coefficients (r) were calculated to explore the relationships between anthropometric variables (height, body mass, and BMI) and performance outcomes (NMAT, NMAT-B, Bangsbo, and Bangsbo-B). The strength of the correlations was interpreted as trivial (*r* < 0.1), small (0.1–0.29), moderate (0.3–0.49), large (0.5–0.69), very large (0.7–0.89), and nearly perfect (≥0.9) (Hopkins et al., 2009). Data were analyzed using the Statistical analyses were performed by JAMOVI software version 2.5 for Macintosh, and the statistical significance was set at *p* < 0.05.

## Results

**Figure 1:**
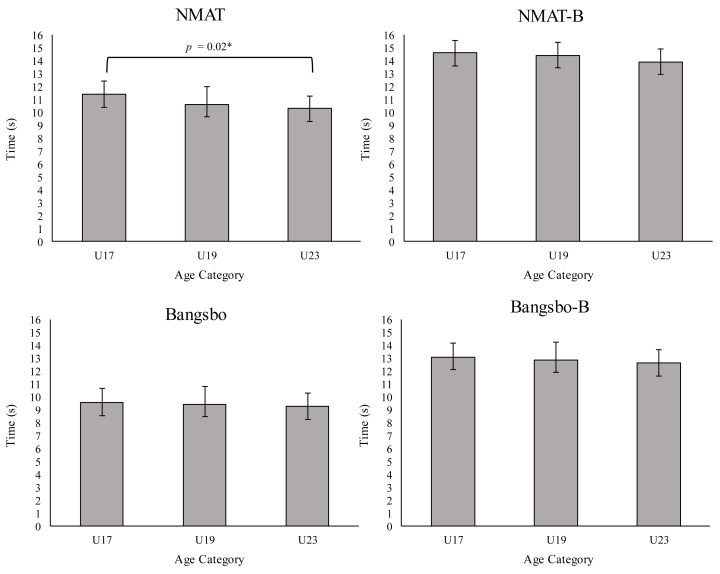
Fitness battery tests according to age groups. NMAT, New Multi-Change of Direction Agility without ball; NMAT-B, New Multi-Change of Direction Agility without ball; Bangsbo, Bangsbo RSA test without ball; Bangsbo-B, Bangsbo RSA test with ball; U17, Under-17; U19, Under-19; U23, Under-23; *Significant level was set at *p* < 0.05.

The differences in the anthropometric measurements and battery fitness test for each age group are presented in Table 1 and Fig. 1, respectively.

### Anthropometric measurements

The repeated measures ANOVA applied to the anthropometric variables showed a significant interaction with age group (F_2.90_,_10__0.2_ = 9.90, *p* <  0.001, *η*^2^_p_ = 0.22). In addition, the between-subjects analysis revealed considerable differences between age groups in global anthropometric measures (F_2_,_6__9_ = 5.48, *p* = 0.006, *η*^2^_p_ = 0.14). In particular, the U23 group showed remarkably greater height than the U17 (*p* = 0.003, ES = 1.12, moderate) and U19 (*p* = 0.01, ES = 0.14, trivial) groups. Regarding body mass, statistically significant differences were also found, with a progressive increase from group U17 to U23. The U17 group showed noticeably lower values with respect to U19 (*p* = 0.04, ES = 0.88, moderate) and U23 (*p* = 0.01, ES = 0.99, moderate), although no significant differences were observed between U19 and U23 (*p* = 1.00). Finally, with respect to BMI, no significant differences were identified between groups (*p* >0.05). The U19 group presented the highest BMI values, while U17 showed the lowest values. Mauchly’s test indicated that the assumption of sphericity was violated (W = 0.62, *p* < 0.001), and Greenhouse-Geisser correction (*ɛ* = 0.73) was applied. Levene’s test revealed homogeneity of variances across age groups for height (*p* = 0.64), body mass (*p* = 0.53), and BMI (*p* = 0.86).

#### NMAT

ANOVA (*F*_1,69_ = 471.43, *p* <  0.001, *η*^2^_p_ = 0.87) showed considerable differences when comparing the results obtained in the NMAT with ball and without ball, indicating that the participants carried out a slower performance during the NMAT-B. When comparing test performance with age groups, no significant difference was observed between test type and age group (*F*_2,69_ = 1.19, *p* = 0.31, *η*^2^_p_ = 0.03). However, noticeable differences were indeed found (*F*_2,69_ = 6.04, *p* = 0.004, *η*^2^_p_ = 0.15) between age groups in overall NMAT performance without a ball with a relative advantage (*p* = 0.02, ES = 1.04 moderate) of the U23 age group (10.30s ± 1.00s) compared to U17 (11.41s ±1.06s). The assumption of sphericity was not violated, as the repeated measures factor had only two levels. Levene’s test confirmed the homogeneity of variances for both test conditions: with ball (*p* = 0.09) and without ball (*p* = 0.16).

**Table 1:** Anthropometric measurements according to age groups.

Variables	Mean ± SD			Pairwise comparisons (*p;* ES)		
	U17	U19	U23	U17 *vs.* U19	U17 *vs.* U23	U19 *vs.* U23
Height (m)	1.69 ± 0.08	1.70 ± 0.07	1.78 ± 0.07	1.00; 0.12	^**^0.01; 1.12	^**^0.01; 1.14
Body Mass (kg)	68.53 ± 5.05	72.99 ± 4.15	73.56 ± 4.45	^*^0.04; 0.88	^**^0.01; 0.99	1.00; 0.14
BMI (kg/m^2^)	24.11 ± 2.49	25.48 ± 2.70	23.44 ± 2.28	1.00; 0.55	1.00; 0.27	0.22; 0.75

**Notes.**

U17 Under-17 U19 Under-19 U23 Under-23

* Significant level was set at *p* < 0.05.

** Significant level was set at *p* < 0.01.

*** Significant level was set at *p* < 0.001.

#### Bangsbo RSA test

The repeated measures ANOVA showed significant differences between the ball and no-ball conditions (F_1,69_ = 998.76, *p* < 0.001, *η*^2^_*p*_ = 0.93), with slower performance when the ball was used. No significant differences were found in the interaction between test type and age group (F_2,69_ = 0.172, *p* = 0.843, *η*^2^_p_ = 0.01). There were also no significant differences between age groups in the overall performance on the Bangsbo RSA Test (F_2,69_ = 1.71, *p* = 0.19, *η*^2^_p_ = 0.05). Similarly, sphericity was assumed due to the two-level structure of the repeated factor. Levene’s test showed homogeneity of variances across age groups for both conditions: with ball (*p* = 0.94) and without ball (*p* = 0.25).

#### Correlations between anthropometric measurements and battery fitness test

Pearson’s correlation analysis revealed moderate negative associations between height and COD performance both without (NMAT: r = −0.35, *p* = 0.01), and with the ball (NMAT-B: r = −0.24, *p* = 0.04). BMI showed small positive correlations with NMAT (*r* = 0.26, *p* = 0.03) and NMAT-B (*r* = 0.24, *p* = 0.04). No noticeable correlations were found between anthropometric variables and Bangsbo RSA performance (*p* >0.05). These results are presented in Table 2.

**Table 2:** Correlations between anthropometric variables and performance.

Variables	Height (r;*p*)	Body Mass (r;*p*)	BMI (r;*p*)
NMAT	−0.35^**^; 0.01	−0.04; 0.75	0.26^*^; 0.03
NMAT-B	−0.24^*^; 0.04	0.06; 0.64	0.24^*^; 0.04
Bangsbo	−0.14; 0.23	0.03; 0.78	0.14; 0.23
Bangsbo-B	−0.06; 0.60	0.01; 0.99	0.05; 0.66

**Notes.**

NMAT New Multi-Change of Direction Agility without ball NMAT-B New Multi-Change of Direction Agility without ball Bangsbo Bangsbo RSA test without ball Bangsbo-B Bangsbo RSA test with ball

* Significant level was set at *p* < 0.05.

** Significant level was set at *p* < 0.01.

*** Significant level was set at *p* < 0.001.

## Discussion

This study aimed to investigate age-related differences in anthropometric characteristics, COD performance and RSA ability, with and without ball control, in elite soccer players from U17, U19, and U23 categories. Based on previous evidence (Borges et al., 2023; Loturco et al., 2020; Silva et al., 2025), it was hypothesized that players in the U23 category would display superior performance in COD and RSA tests as compared to U17 and U19 players, with the greatest differences expected in the no-ball condition, where technical constraints are minimal and physical, anthropometric, and training factors may dominate. The main findings partially supported this hypothesis: U23 players were, on average, taller and heavier than U17 and U19 players, and showed superior COD performance without the ball when compared to U17. However, differences between U19 and other groups were minor and not statistically significant, and no significant age-related differences were observed in the Bangsbo RSA test.

The anthropometric results align with previous research showing that increases in height and body mass are natural outcomes of age-related anthropometric characteristics during adolescence, and these traits are closely associated with strength development and performance in high-intensity actions (Borges et al., 2023; Lloyd & Oliver, 2012). Greater body size in older players may support improved power generation during accelerations and decelerations, which are key determinants of COD efficiency. However, body composition rather than absolute body mass remains critical for optimal performance, as excess fat mass may impair agility and repeated sprinting ability (França et al., 2024; Stanković et al., 2023). Specifically, França et al. (2024), who examined 96 youth male soccer players aged 13–19 years and found that lower-body power and fat-free mass were strongly associated with sprint and agility performance (*r* = −0.65 to 0.69). Although the testing protocols differ, the magnitude of the effects observed in their study is comparable to the age-related differences found in this work’s COD analyses (*η*^2^_p_ = 0.22). Therefore, both studies point to a consistent quantitative trend: as players grow in terms of anthropometry and age, agility-related performance improves substantially, regardless of the specific test used. These findings emphasize the notion that anthropometric monitoring should accompany technical and physical assessments to guide individualized training.

Furthermore, the correlation analysis confirmed that anthropometric variables were moderately associated with COD performance. Specifically, taller players showed better NMAT (*r* = −0.35) and NMAT-B (*r* = −0.24) results, likely due to advantages in stride length, momentum, and acceleration during direction changes (Borges et al., 2023; Campa et al., 2019). In contrast, higher BMI values were related to slower performance in COD tests (*r* = 0.26 to 0.24), possibly reflecting the negative effect of excess body mass on movement efficiency (Silva et al., 2016). These findings agree with previous literature suggesting that anthropometric characteristics such as stature and BMI play a role in agility performance yet have a limited influence on repeated sprint ability (Borges et al., 2023; França et al., 2024; Stanković et al., 2023). For example, França et al. (2024) showed that age or body composition could explain between 22% of the variance observed in agility scores, which is consistent with the magnitude of COD differences observed between the sampled U17 and U23 groups.

Regarding COD performance, the results highlight that NMAT without a ball improved significantly with age, especially among U17 and U23 players, suggesting that age-related and anthropometric factors may contribute to differences in COD efficiency. These findings are consistent with studies indicating that older youth players display superior agility due to better neuromuscular coordination, greater muscle strength, and refined motor control (Lerche et al., 2024; Trecroci et al., 2020; Young & Farrow, 2006). Popowczak et al. (2020) also identified age (*β* = −0.30) and training experience (*β* = −0.18) as significant predictors of COD performance. However, when the ball was introduced, age-related improvements were not significant, suggesting that technical skill does not progress at the same rate as somatic stature. This is in line with studies emphasizing that dribbling proficiency and decision-making require deliberate, sport-specific practice rather than being automatic consequences of age-related anthropometric characteristics (Forsman et al., 2016; Sal de Rellán-Guerra et al., 2019; Thieschäfer & Büsch, 2022). Likewise, training experience (*β* = −0.18) has been identified as a predictor of COD performance (Popowczak et al., 2020). For coaches, this highlights the need to integrate COD drills with ball control into training from early ages to ensure technical skills evolve alongside physical development.

In contrast, no significant age-related differences were found in Bangsbo RSA performance, despite a trend toward improvement with age. This suggests that RSA may plateau earlier in development or be less sensitive to age-related changes compared to COD. Previous research supports that RSA depends on multifactorial aspects such as metabolic capacity, recovery efficiency, and tactical behaviours, which may not differ markedly between players of closely related age groups (Girard, Mendez-Villanueva & Bishop, 2011; Kyles et al., 2023; López-Segovia et al., 2014; Thurlow et al., 2023). Similar studies also reported that sprinting, anaerobic power, and maximal strength do not always differ significantly between U17 and U20 players (Kobal et al., 2016; Nikolaidis et al., 2016). In addition, young soccer players can achieve similar results in Bangsbo RSA Test, after a training programme (Jorge, Garrafoli & Cal Abad, 2020). These findings may suggest that improvement in these tests depends not as much on age as on their training.

Overall, these results seem to indicate that age-related anthropometric differences and general physical development may contribute to improved COD performance, particularly without the ball, whereas technical proficiency and RSA appear to depend more on specific training stimuli than on chronological age. This underscores the importance of adopting an integrated approach in player development, where physical, technical, and tactical capacities are trained simultaneously. Despite its valuable insights, this study has some limitations. The sample size was limited to 72 players across three elite soccer clubs, which may affect the generalizability of findings. Additionally, the study focused primarily on agility and anthropometric measurements, without considering muscle strength, power, body fat percentage, or positional differences, which could further influence COD performance (França et al., 2022; França et al., 2024). Future research should explore longitudinal designs and include physiological markers (*e.g.*, VO_2_max, lactate thresholds) to better understand the interplay between growth, training, and performance adaptations across developmental stages.

## Conclusions

This study examined age-related differences in COD and RSA performance with and without ball control in U17, U19, and U23 elite soccer players. The hypothesis that older players would outperform younger ones was only partially supported. Specifically, U23 players showed superior COD performance without the ball compared to U17 players, while COD with ball and RSA performance did not differ significantly across age groups. These results indicate that age-group differences are reflected mainly in tasks with lower technical constraints, while performance in tests requiring ball control or repeated sprints appears to be more influenced by specific training adaptations than by chronological age.

The major contribution of this study is the simultaneous assessment of COD and RSA with and without ball control, highlighting the fact that technical demands constrain performance across all developmental stages. The additional correlation analyses revealed that certain anthropometric traits, such as height and BMI, were moderately associated with COD performance, which suggests that physical characteristics may contribute to age-group differences in agility. In contrast, RSA performance showed no significant relationship with anthropometric variables, reinforcing the idea that this ability likely depends more on metabolic and neuromuscular conditioning than on body structure.

Overall, these findings highlight the need for training programmes that integrate physical, technical, and tactical components across all age categories, ensuring that improvements in agility and sprint capacity are accompanied by parallel progress in ball-handling and decision-making skills.

##  Supplemental Information

10.7717/peerj.20691/supp-1 Supplemental Information 1 STROBE checklist

10.7717/peerj.20691/supp-2 Supplemental Information 2 Raw data

## Additional Information and Declarations

### Data Availability

The following information was supplied regarding data availability: The data is available in the Supplementary File.
