# Supplementary material for: Change of direction and Repeated Sprint Ability with and without ball performance in young soccer players: a comparison across different age-categories
Source: PeerJ. 2026 Feb 2;14:e20691. doi: 10.7717/peerj.20691 (PMC12875218; doi:10.7717/peerj.20691)
Supplement: Supplemental Information 1 [file peerj-14-20691-s001.pdf]

STROBE Statement—checklist of items that should be included in reports of observational studies

	Item No.	Recommendation	Page No.	Relevant text from manuscript
**Title and abstract**	1	(*a*) Indicate the study's design with a commonly used term in the title or the abstract	1	The title indicates the study design ("comparison across different age-categories").
		(*b*) Provide in the abstract an informative and balanced summary of what was done and what was found	1	The abstract summarizes background, methodology, results, and conclusions
**Introduction**				
Background/rationale	2	Explain the scientific background and rationale for the investigation being reported	2-3	Context and importance of studying COD and RSA in youth soccer players.
Objectives	3	State specific objectives, including any prespecified hypotheses	3	At the end of the introduction, the objective of the project is clearly defined: to investigate age-related differences in anthropometric traits, COD, and RSA.
**Methods**				
Study design	4	Present key elements of study design early in the paper	4	Cross-sectional design described: Methods, Measures.
Setting	5	Describe the setting, locations, and relevant dates, including periods of recruitment, exposure, follow-up, and data collection	4-5	Data collection setting, time frame, training/match season, and environmental conditions.
Participants	6	(*a*) *Cohort study*—Give the eligibility criteria, and the sources and methods of selection of participants. Describe methods of follow-up<br>*Case-control study*—Give the eligibility criteria, and the sources and methods of case ascertainment and control selection. Give the rationale for the choice of cases and controls<br>*Cross-sectional study*—Give the eligibility criteria, and the sources and methods of selection of	3-4	Eligibility criteria, recruitment, sample size per group, informed consent, and IRB approval in "participants"

		participants		
		(*b*) *Cohort study*—For matched studies, give matching criteria and number of exposed and unexposed<br>*Case-control study*—For matched studies, give matching criteria and the number of controls per case		
Variables	7	Clearly define all outcomes, exposures, predictors, potential confounders, and effect modifiers. Give diagnostic criteria, if applicable	6-7	Definitions of all outcomes and exposures (age, height, weight, BMI, COD, RSA)
Data sources/ measurement	8*	For each variable of interest, give sources of data and details of methods of assessment (measurement). Describe comparability of assessment methods if there is more than one group	5	The entire data collection procedure and description of the tests and instruments can be found in the "Design and Procedures" section. Instruments and procedures (stadiometer, electronic scale, infrared photoelectric cells NMAT, Bangsbo RSA)
Bias	9	Describe any efforts to address potential sources of bias	4	Steps to minimize bias (familiarization, standardized conditions, same testers)
Study size	10	Explain how the study size was arrived at	3-4	Sample size justification (n=72, equally divided into U17, U19, U23): Methods, Participants,

Continued on next page

Quantitative variables	11	Explain how quantitative variables were handled in the analyses. If applicable, describe which groupings were chosen and why	5-6	How quantitative variables were handled (mean ± SD, a repeated measure ANOVA, effect sizes): Methods, Statistical Analysis
Statistical methods	12	(*a*) Describe all statistical methods, including those used to control for confounding	5-6	Tests used: Shapiro-Wilk, Levene, repeated measures ANOVA, Bonferroni, Cohen's d, Jamovi software: Methods, Statistical Analysis
		(*b*) Describe any methods used to examine subgroups and interactions		
		(*c*) Explain how missing data were addressed		
		(*d*) *Cohort study*—If applicable, explain how loss to follow-up was addressed<br>*Case-control study*—If applicable, explain how matching of cases and controls was addressed<br>*Cross-sectional study*—If applicable, describe analytical methods taking account of sampling strategy		
		(*e*) Describe any sensitivity analyses		
**Results**				
Participants	13*	(a) Report numbers of individuals at each stage of study—eg numbers potentially eligible, examined for eligibility, confirmed eligible, included in the study, completing follow-up, and analysed	4-5	Flow and numbers of participants reported (72 players across 3 groups)
		(b) Give reasons for non-participation at each stage		
		(c) Consider use of a flow diagram		
Descriptive data	14*	(a) Give characteristics of study participants (eg demographic, clinical, social) and information on exposures and potential confounders	6-7	The differences in the anthropometric measurements and battery fitness test to each age group are presented in Table 1 and results section
		(b) Indicate number of participants with missing data for each variable of interest		
		(c) *Cohort study*—Summarise follow-up time (eg, average and total amount)		
Outcome data	15*	*Cohort study*—Report numbers of outcome events or summary measures over time		
		Case-control study—Report numbers in each exposure category, or summary measures of exposure		

		Cross-sectional study—Report numbers of outcome events or summary measures	6-7	Group differences in COD and anthropometric measurements
Main results	16	(*a*) Give unadjusted estimates and, if applicable, confounder-adjusted estimates and their precision (eg, 95% confidence interval). Make clear which confounders were adjusted for and why they were included	6-7	Comparisons with and without ball, age interactions. Table 1.
		(*b*) Report category boundaries when continuous variables were categorized		
		(*c*) If relevant, consider translating estimates of relative risk into absolute risk for a meaningful time period		

Continued on next page

Other analyses	17	Report other analyses done—eg analyses of subgroups and interactions, and sensitivity analyses		
**Discussion**				
Key results	18	Summarise key results with reference to study objectives	7	Main findings restated and compared with objectives: First paragraph discussion
Limitations	19	Discuss limitations of the study, taking into account sources of potential bias or imprecision. Discuss both direction and magnitude of any potential bias	8	The sample size was limited to 72 players across three elite soccer clubs, which may affect the generalizability of findings. Additionally, the study focused primarily on agility and body composition, without considering muscle strength, power, or positional differences, which could further influence COD performance. Future research should explore longitudinal designs and include physiological markers (e.g., VO_2max, lactate thresholds) to better understand the interplay between growth, training, and performance adaptations across developmental stages: Last paragraph discussion
Interpretation	20	Give a cautious overall interpretation of results considering objectives, limitations, multiplicity of analyses, results from similar studies, and other relevant evidence	7-8	Relation to previous literature and practical implications
Generalisability	21	Discuss the generalisability (external validity) of the study results	8	Applicability of findings to training and player development
**Other information**				
Funding	22	Give the source of funding and the role of the funders for the present study and, if applicable, for the original study on which the present article is based	9	Mehdi Ben Brahim received funding for the publication fees for

			this work from Prince Sultan University. The funders had no role in study design, data collection and analysis, decision to publish, or preparation of the manuscript.

*Give information separately for cases and controls in case-control studies and, if applicable, for exposed and unexposed groups in cohort and cross-sectional studies.

Note: An Explanation and Elaboration article discusses each checklist item and gives methodological background and published examples of transparent reporting. The STROBE checklist is best used in conjunction with this article (freely available on the Web sites of PLoS Medicine at http://www.plosmedicine.org/, Annals of Internal Medicine at http://www.annals.org/, and Epidemiology at http://www.epidem.com/). Information on the STROBE Initiative is available at www.strobe-statement.org.
